# The regulatory effect of has-circ-0001146/miR-26a-5p/MNAT1 network on the proliferation and invasion of osteosarcoma

**DOI:** 10.1042/BSR20201232

**Published:** 2020-06-09

**Authors:** Junjie Wang, Jiangdong Ni, Deye Song, Muliang Ding, Jun Huang, Wenzhao Li, Guangxu He

**Affiliations:** Department of Orthopedics, The Second Xiangya Hospital of Central South University, Changsha 410011, China

**Keywords:** has-circ-0001146, miR-26a-5p, MNAT1, osteosarcoma

## Abstract

Osteosarcoma is a malignant bone tumour with the lowest survival rates out of all paediatric cancers and is primarily diagnosed in children and adolescents. MNAT1 is a subunit in the cyclin-dependent kinase-activating kinase complex. Abnormal up-regulation of MNAT1 has been associated with the poor prognosis of multiple cancers. Bioinformatics analysis showed that has-circ-0001146 and miR-26a-5p were involved in the regulation of MNAT1 in osteosarcoma. The present study investigated the regulatory effects of has-circ-0001146 and miR-26a-5p on MNAT1 expression using luciferase reporter and RNA-pull down assays. The effects of the has-circ-0001146/miR26a-5p/Mnat1 network on the proliferation and invasion of osteosarcoma were evaluated by cell viability, apoptosis, migration, and invasion assays. Osteosarcoma tissues showed higher MNAT1 and has-circ-0001146 expression than adjacent normal tissues, although the expression of MNAT1 was not significantly up-regulated in sarcomas according to TCGA databases. As indicated by luciferase reporter and RNA-pull down assays, miR-26a-5p was able to bind to both has-circ-0001146 and *MNAT1* mRNA. The depletion of has-circ-0001146 as well as the increase of miR-26a-5p decreased MNAT1 expression in osteosarcoma cells, while the reduction of miR-26a-5p was associated with increased MNAT1 expression. These data suggested that has-circ-0001146 promoted MNAT1 expression by competitively binding to miR-26a-5p with *MNAT1* mRNA. The depletion of has-circ-0001146 or MNAT1 or the increase of miR-26a-5p inhibited osteosarcoma cell viability and invasion, and increased apoptosis. Reduction of miR-26a-5p conversely promoted osteosarcoma cell viability and invasion. The present study confirmed that has-circ-0001146 blocked miR-26a-5p targeting MNAT1 in osteosarcoma cells, thereby promoting the malignant behaviours of osteosarcoma cells.

## Introduction

Osteosarcoma (OS) is a malignant bone tumour common in children and adolescents. Worldwide, the incidence of OS is increasing annually and accounts for approximately 35% of primary malignant bone tumours [1,2]. OS has one of the lowest survival rates out of all paediatric cancers despite the use of multiple chemotherapeutic regimens before and after surgery [1,2]. Early distant metastasis, especially extensive lung metastasis, is one of the primary causes of death in OS patients. The long-term disease-free survival rate of OS patients is still less than 50% [1–3]. A new investigation in China found that approximately 12.6% of patients already had distant metastasis at the time of diagnosis, approximately 9% of patients have local recurrence within 3 years of treatment, and more than 30% of patients have distant organ metastasis after treatment [3,4]. The pathogenesis of OS has not been fully elucidated, which hinders the improvement of OS treatment. Therefore, it is important to clarify the pathogenesis of OS and seek appropriate treatment measures to improve patient prognosis and quality of life.

MNAT1 (menage a trois 1, MAT1) is the third subunit in cyclin-dependent kinase-activating kinase (CAK) complex in addition to CDK7 and Cyclin H [5]. MNAT1 serves as an assembly factor and a substrate specificity-determining factor of CAK to promote the stability and activation of CAK [6]. Activated CAK phosphorylates cell cycle regulators such as CDKs and retinoblastoma tumour suppressor protein, which promotes cell proliferation and activates a series of transcription factors (including p53, Octs, retinoic acid receptor α (RARα), and peroxisome proliferator-activated receptor γ (PPARγ), thereby regulating gene transcription [5,6]. Abnormal up-regulation of MNAT1 has been observed in breast cancer and colorectal cancer in recent studies and is associated with rapid cancer malignance and poor prognosis [5,6].

Non-coding RNAs (ncRNAs) are a group of RNAs that are not translated into proteins but play an important role in the epigenetic regulation of gene expression. ncRNAs mainly include tRNAs, rRNAs, miRNAs, siRNAs, lncRNAs, and circRNAs [7,8]. MicroRNAs (miRNAs) are abundant and functionally important; they are ∼19–25 nucleotides in length. miRNAs are involved in multiple cellular functions via the post-transcriptional regulation of gene transcription [7,8]. According to previous studies, the involvement of several miRNAs in the development of OS and other cancers has been consistently reported [7–9]. Circular RNAs (circRNAs) are also a vital ncRNA family member. They are characterized by a covalently closed continuous loop without 5′ caps and 3′ poly(A) tails [7,8]. Due to the special circular structure, circRNAs are resistant to RNase R digestion; this makes circRNAs more stable than their linear counterparts. circRNAs can function as competitive endogenous RNAs (ceRNAs) to sponge miRNAs, thus impairing the regulatory effect of miRNAs on the targeted mRNA [7,8]. Based on this specialized function of circRNAs, many studies have confirmed that the interactions between circRNAs and miRNA are involved in the development of many cancers.

Previous studies using Microarray and high-throughput sequencing technologies found many ncRNAs that were aberrantly expressed in OS cancer tissues compared with adjacent normal tissues [10–12]. By bioinformatics analysis, we further found that potential interaction between has-circ-0001146 and miR-26a-5p among these ncRNAs, and their interaction, might influence the of Mnat1 expression. The present study aimed to identify the interaction among has-circ-0001146/miR26a-5p/Mnat1 and further investigate the effect of this regulatory network on the proliferation and invasion of OS cells.

## Materials and methods

### OS cancer samples

A total of 21 paired OS cancer and adjacent normal tissues were obtained from patients undergoing OS surgery at Xiangya hospital (Changsha, China) between 2016 and 2018. Samples of tumour tissue and surrounding morphologically normal tissue, taken from the tumour (>3 cm), were obtained from each patient. Patient clinical data are presented in Table 1. Patients were excluded from the present study if they had received prior radiotherapy, systemic venous chemotherapy, or immunotherapy. All tissue specimens were preserved at −80°C following surgical resection. Informed consent was obtained from all patients and the study protocol was approved by Xiangya hospital. The present study was performed in accordance to the Declaration of Helsinki.

**Table 1:** Clinicopathological characteristics of patients with OS

	Case (*n*)
Gender	
Male	11
Female	10
Age, years	
<21	19
≥21	2
Tumor size, cm^3^	
<2	16
≥2	5
Clinical stage	
Stage I/II	13
Stage III/IV	9
Metastasis	
Yes	4
No	17

### PCR assay

Total RNA was extracted from the frozen tissues and cultured cells using an RNeasy kit (Qiagen, Shanghai, China) according to the manufacturer’s instructions. cDNA was prepared by reverse transcription of total RNA using a High Capacity RNA-to-cDNA Master Mix (Qiagen). RT-qPCR was performed using the SYBR ExScript RT-PCR kit (TaKaRa, Dalian, China) on an ABI 7300 Real-Time PCR System (Applied Biosystems, Foster City, CA, U.S.A.) according to manufacturer’s instructions. The thermocycling profile was as follows: 95°C for 30 s, followed by 40 cycles of 95°C for 5 s and 60°C for 30 s. The primer sequences are shown in Table 2. Gene expression was normalized to GAPDH expression using the 2^−ΔΔCT^ method.

**Table 2:** The sequences of primers used for PCR assay

Gene name	Primer orientation	Sequences
hsa-circ-0001146	Forward	5′-GTCTTGCTCAGTTGCCCAGG-3′
	Reverse	5′-AAGTCAATCCTGTGGGCAAC-3′
miR-26a-5p	Forward	5′-UCCAUAAAGUAGGAAACACUACA-3′
	Reverse	5′-CAGUACUUUUGUGUAGUACAA-3′
MNAT1	Forward	5′-GGTTGCCCTCGGTGTAAGAC-3′
	Reverse	5′-AGTTGCTCTTTCTGAGTGGAGT-3′
GAPDH	Forward	5′-GGGTGTGAACCATGAGAAGT-3′
	Reverse	5′-TGAGTCCTTCCACGATACCAA-3′
U6	Forward	5′-CTCGCTTCGGCAGCACA-3′
	Reverse	5′-AACGCTTCACGAATTTGCGT-3′

### Western blotting

The frozen tissues and cultured cells were lysed with lysis buffer [1 × PBS, 1% Nonidet P-40, 0.5% sodium deoxycholate, 0.1% SDS, and freshly added 100 μg/ml phenylmethanesulfonyl fluoride (PMSF), 10 μ g/ml aprotinin, and 1 mM sodium orthovanadate]. The protein concentration of the lysates was measured using the BCA Reagent Kit (Beyotime) according to manufacturer’s guidelines. Equal amounts of the protein samples (20 μg) were separated by 10% SDS-PAGE and then transferred onto a nitrocellulose membrane. The blot was blocked with 5% non-fat milk, incubated with the anti-MNAT1, ant-Ki67 and anti-GAPDH antibodies (Dilution 1:1000, Abcam, Cambridge, U.K.) at 4°C overnight, followed by incubation with an appropriate peroxidase conjugated secondary antibody (Vector Laboratories lnc, Burlingame, CA, U.S.A.). The signal was developed using 4-chloro-1-napthol/3, 3-o-diaminobenzidine, and relative photographic density was quantified by a gel documentation and analysis system. GAPDH was used as an internal control to verify basal expression levels and equal protein loading. The ratio of the specific proteins to GAPDH was calculated.

### Cell culture

OS cell lines, MG-63, and 143B were obtained from American Type Culture Collection (ATCC, Manassas, VA, U.S.A.). All the cell lines were grown in DMEM supplemented with 10% foetal bovine serum (FBS) (Invitrogen, Grand Island, NY, U.S.A.) and 1% antibiotic-antimycotic (Invitrogen). Cells were maintained in a 5% CO_2_ atmosphere at 37°C in a humidified tissue culture incubator.

### Cell transfection

Short hairpin (sh) RNAs target MNAT1 and has-circ-0001146 were constructed by GenePharma (Shanghai, China). MG-63 and 143B cells were seeded onto six-well plates with a density of 1 × 10^5^ cells/well. The cells were transfected with shRNA-MNAT1, shRNA-has-circ-0001146 and scrambled shRNA using Lipofectamine 2000 (Invitrogen, Waltham, Massachusetts, U.S.A.), according to manufacturer’s protocols. miR-26a-5p mimics and inhibitors (GenePharma) were synthesized to increase and decrease miR-26a-5p expression in OS cells, respectively. miR-26a-5p mimics and inhibitors were transfected into MG-63 and 143B cells using Lipofectamine 2000 (Invitrogen). The expression levels of has-circ-0001146, miR-26a-5p and MNAT1 were assessed 12 h after the transfection using PCR assay.

### RNA-pull down

Biotinylated RNA probes (Bio-miR-NC, Bio-miR-26a-5p-WT and Bio- miR-26a-5p-Mut) were incubated with the lysates of 143B cells and extracted by means of streptavidin-coupled magnetic beads according to the instructions for the Pierce™ Magnetic RNA Pull-Down Kit (Rockford, IL, U.S.A.). RNA–RNA complexes were then eluted with the salt solution and purified using TRIzol (Pierce). The enrichment of has-circ-0001146 in the RNA–RNA complexes was quantified by qPCR, as described above.

### Luciferase reporter assay

Bioinformatics analysis by Starbase (http://starbase.sysu.edu.cn/index.php) identified a putative binding site on 3′UTR of MNAT1 gene. A wild-type sequence containing this binding site was established by PCR. In addition, the binding site was mutated in the wild-type sequence to establish the mutant sequence. The wild-type and mutant sequences were cloned into the pGL3 vector. The vectors were transfected into 143B cells alone or with miR-26a-5p mimics using Lipofectamine 2000 (Invitrogen). Cells were harvested at 24 h and the activity of firefly luciferase was normalized to that of Renilla luciferase.

### Fluorescence *in situ* hybridization (FISH)

FISH assay was employed to determine the localization of has-circ-0001146 in OS cells. The has-circ-0001146 probe was synthesized by RiboBio, Co., Ltd., (Guangzhou China). 143B cells were incubated with has-circ-0001146 probe overnight at 37°C. After washed with Phosphate-Buffered Saline/Tween (PBST) three times, the cells were further stained with 4′,6-Diamidino-2-Phenylindole (DAPI) at the ratio of 1: 800. The images of cells were captured using the fluorescence microscope (Olympus Optical Co., Ltd., Tokyo, Japan).

### MTT assay

Cell viability was determined by performing MTT [3-(4,5-dimethyl-2-thiazolyl)-2,5-diphenyl-2-H-tetrazolium bromide] assays (add company name for MTT). Briefly, cells (1 × 10^3^) were seeded in 96-well microplates. The cells were cultured for the indicated time, followed by incubation with MTT for 4 h at 37°C. Optical density (OD) was determined at 450 nm using a microplate reader.

### Cell apoptosis assay

MG-63 and 143B cells were harvested and washed with cold phosphate-buffered saline (PBS), and then stained with 5 μl of AnnexinV-FITC (KeyGen Biotech, Shanghai, China) and 10 μl of PI (BD Pharmingen, San Diego, CA, U.S.A.) in the dark. The number of apoptotic cells was quantified by flow cytometry (BD Biosciences, San Jose, CA, U.S.A.).

### Wound-healing assay

The wound-healing assay was used to investigate cell migration. A 1-ml pipette tip was used to create a ‘scratch-wound’ on the MG-63 and 143B cell monolayer. The culture medium was then replaced with FBS-free medium. Microscope images of the cells were captured immediately following scratching and after 24 h. The cell migration rate was calculated based on the movement of cells from initial placement to the final distance travelled following 24 h. Cell migration rate was calculated using the following equation:

$$\frac{\text{(initial distance-final distance)}}{\text{initial distance}}\times 100$$

### Transwell assay

MG-63 and 143B cells were cultured in medium without serum in the upper chamber of a trans-well (24-well insert, 8-mm pore size, Millipore). The upper chamber was coated with Matrigel (Sigma-Aldrich; Merck KGaA). Complete medium was subsequently added to the lower chamber. Following culture for 24 h, non-invading cells on the upper surface of the membrane were removed with a cotton-tipped swab, while the invading cells on the surface of the lower chamber were stained with 0.1% Crystal Violet at room temperature for 20 min. The invading cells were quantified by counting 10 random fields of view at ×200 magnification using a light microscope (E200; Nikon Corporation, Tokyo, Japan)

### Growth of xenograft tumor measument

Male BALB/C nude mice were purchased from Beijing Vital River Laboratory Animal Technology (Beijing, China). Animal experiments strictly followed the guidelines of institutional guidelines of the Research Ethics Committee of the 2nd Xiangya Hospital of Central South University. 143B cells were transfected with a lentivirus containing shRNA-has-circ-0001146 (GenePharma) to induce the stable knockdown of has-circ-0001146. 143B cells (6.0 × 10^6^) with has-circ-0001146 knockdown and not were injected subcutaneously into the back of mice. The tumor volume was calculated every week using the following formula: $\text{V}={\text{lw}}^{2}/2$, where (*l*) is the length, (*w*) the width, and (*V*) is the volume.

### Immunohistochemical analysis

Xenograft tumor tissues were embedded in paraffin. The sections (4-µ thick) were deparaffinized and blocked with 5% BSA for 30 min at room temperature. Afterwards, sections were incubated with anti-Ki67 primary antibody overnight at 4°C and then with the diaminobenzidine (DAB)-conjugated second antibody at room temperature for 10 min. DAB was applied for 5 min, followed by counterstaining with Mayer’s hematoxylin.

### Statistical analyses

Data were analysed in SPSS 12.0 software (SPSS, Inc., Chicago, IL, U.S.A.). One-way analysis of variance followed by Scheffe’s post hoc test was performed to evaluate statistical significance (*P*<0.05).

## Results

### Overexpressed MNAT1 is observed in OS tissues

**Figure 1:**
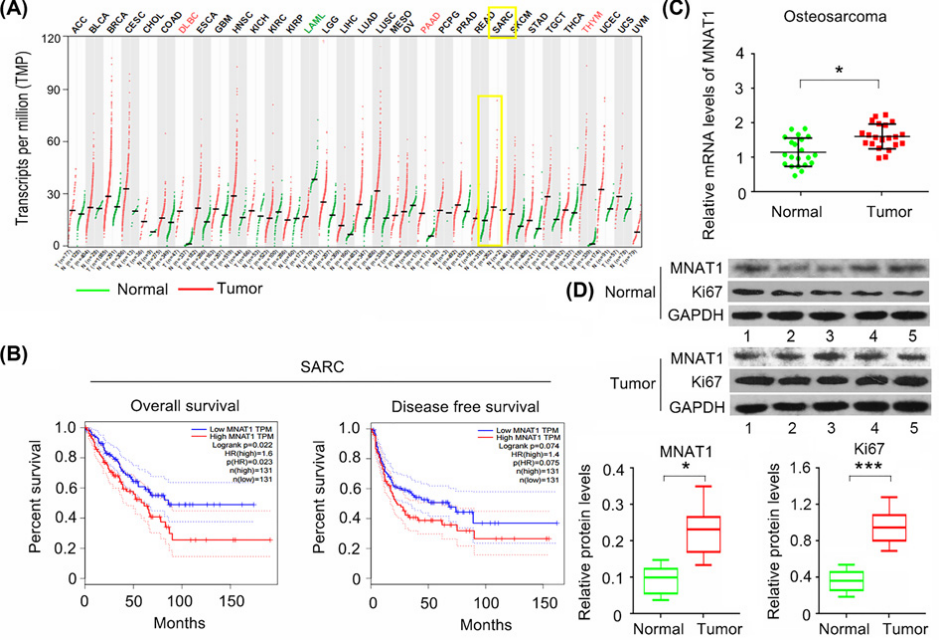
MNAT1 is overexpressed in OS tissues (**A**) The expression of MNAT1 was analysed in various types of cancers using the data in TCGA databases (http://gepia.cancer-pku.cn/). Although the expression of MNAT1 appeared to be up-regulated in sarcoma (SARC), the up-regulation did not reach a statistically significant difference. (**B**) The expression MNAT1 is closely associated with the overall survival ratio of patients with SARC, but not with the disease-free ratio. (**C**) The present study tested the expression of MNAT1 in OS and adjacent normal bone tissues. PCR analysis showed that MNAT1 mRNA level was higher in osteosarcoma than in normal bone tissues. (**D**) Western blot analysis also confirmed the overexpression of MNAT1 and Ki67 proteins in OS. Five representative blots were shown in the Figure. *P<0.05 and ***P<0.001 vs. normal bone tissues.

The present study initially analysed the expression of MNAT1 in various types of cancers using the data in TCGA databases (http://gepia.cancer-pku.cn/). As indicated by Figure 1A, MNAT1 is up-regulated in lymphoid neoplasm diffuse large B-cell lymphoma (DLBC), pancreatic adenocarcinoma (PAAD), in comparison with corresponding normal tissues. Although the expression of MNAT1 appeared to be up-regulated in sarcoma (SARC), the up-regulation did not reach a statistically significant difference. The expression MNAT1 is closely associated with the overall survival ratio, but not with the disease-free ratio (Figure 1B). SARC includes Ewing’s sarcoma, synovial sarcoma, OS and so on. Therefore, the data from the SARC samples may not be an actual representation of pure OS. To resolve this problem, the present study additionally tested the expression of MNAT1 in OS and normal bone tissues. PCR analysis showed that MNAT1 mRNA level was higher in OS than in normal bone tissues (*P*<0.05, Figure 1C). Western blot analysis confirmed the overexpression of MNAT1 protein (*P*<0.05, Figure 1D) and an OS marker Ki67 (*P*<0.001) in OS.

### miR-26a-5p modulated MNAT1 expression in OS

**Figure 2:**
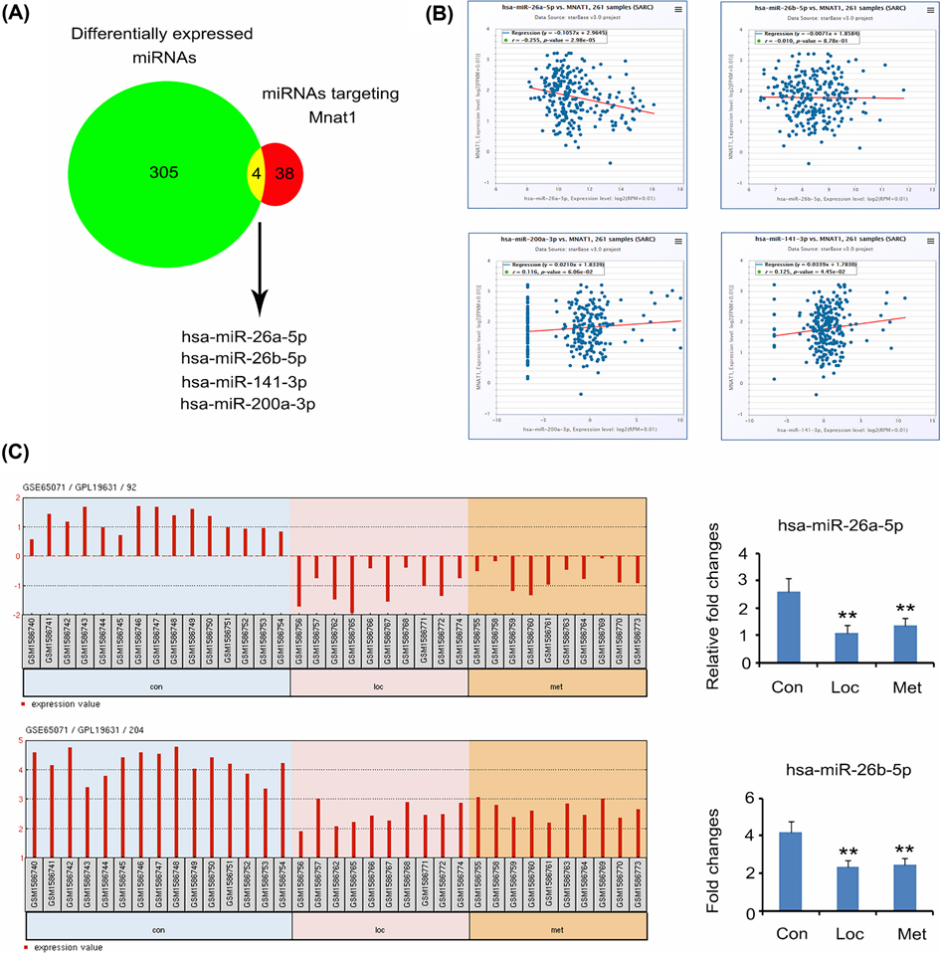
The implication of miR-26a-5p in MNAT1 overexpression in OS (**A**) The present study probed for miRNAs that are both aberrantly expressed in osteosarcoma according to GSE65071 as well as predicted to target MNAT1 (http://www.targetscan.org/vert_72/). Four miRNAs, miRNA-26a-5p, miRNA-26b-5p, miRNA-200a-3p, and miRNA-141-3p, satisfied these two conditions. (**B**) Next, we analysed the correlation between MNAT1 and these four miRNAs in their expression using the data in TCGA databases (http://starbase.sysu.edu.cn/index.php). miRNA-26a-5p expression showed negative correlation with MNAT1 expression. In contrast, miRNA-141-3p expression had positive correlation with MNAT1 expression. No correlation was observed between MNAT1 expression with miRNA-26b-5p and miRNA-200a-3p expression. (**C**) The present study further analysed the expression of miRNA-26a-5p and miRNA-26b-5p in osteosarcoma and corresponding normal tissues using data in the GSE65071 database. Both miRNA-26a-5p and miRNA-26b-5p were lowly expressed in both local and metastatic osteosarcoma. Con: Control tissues, namely normal bone tissues; Loc: local osteosarcoma tissues; Met: metastatic osteosarcoma tissues. **P<0.01 vs. normal bone tissues.

GSE65071 datasets recorded the abnormally expressed miRNAs in OS tissues. To determine the miRNAs that might be responsible for the overexpression of MNAT1 in OS, the present study probed for miRNAs that are both aberrantly expressed in OS, as well as predicted to target MNAT1 (http://www.targetscan.org/vert_72/). Four miRNAs, miRNA-26a-5p, miRNA-26b-5p, miRNA-200a-3p, and miRNA-141-3p, satisfied these two conditions (Figure 2A). Next, we analysed the correlation between MNAT1 and these four miRNAs in their expression using the data in TCGA databases (Figure 2B). miRNA-26a-5p expression showed negative correlation with MNAT1 expression. In contrast, miRNA-141-3p expression had positive correlation with MNAT1 expression. No correlation was observed between MNAT1 expression with miRNA-26b-5p and miRNA-200a-3p expression. The present study further analysed the expression of miRNA-26a-5p and miRNA-26b-5p in OS and corresponding normal tissues using data in the GSE65071 database. Both miRNA-26a-5p and miRNA-26b-5p were lowly expressed in both local and metastatic OS (*P*<0.01, Figure 2C). Based on these findings, miRNA-26a-5p is most likely involved in the up-regulation of MNAT1 expression in OS.

### has-circ-0001146 regulated MNAT1 expression by sponging miR-26a-5p

**Figure 3:**
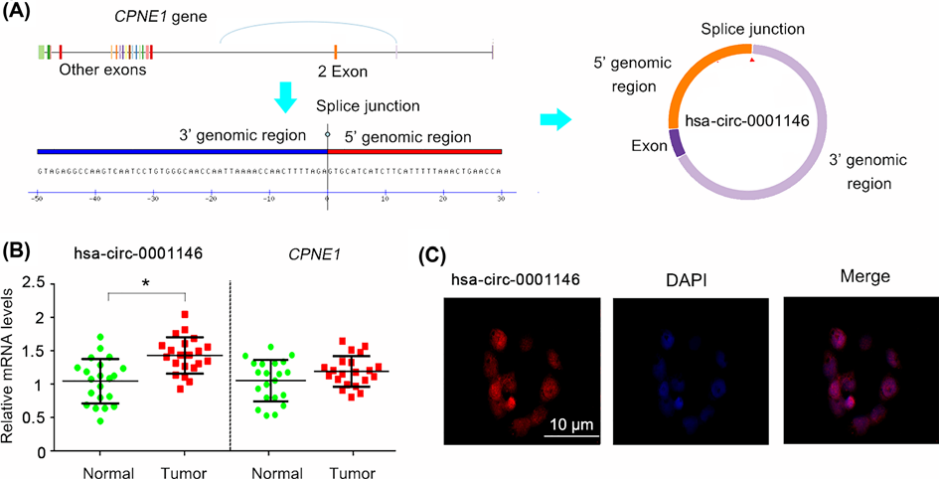
Characters of has-circ-0001146 in OS (**A**) hsa-circ-0001146 is formed by the junction between 5′ and 3′ regions of *CPNE1* mRNA variant 3. hsa-circ-0001146 contains the sequences of Exon 2 and introns at both sides of Exon 2. (**B**) As indicated by PCR assay, hsa-circ-0001146 was up-regulated in OS tissues in comparison with the corresponding normal tissues. *P<0.05 *vs*. normal bone tissues. (**C**) FISH analysis demonstrated that hsa-circ-0001146 was located in both nucleus and cytoplasm of 143B cells.

**Figure 4:**
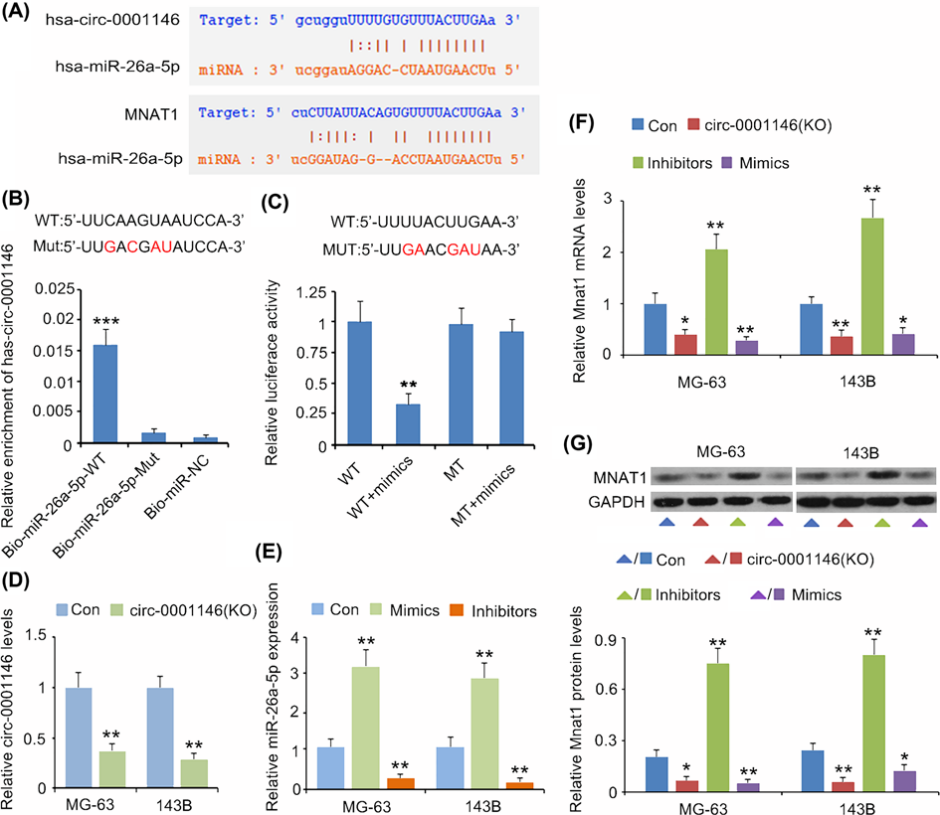
Has-circ-0001146 promoted MNAT1 expression by sponging miR-26a-5p (**A**) Bioinformatics analysis showed that miR-26a-5p can bind to both hsa-circ-0001146 and MNAT1 3′UTR at completely consistent base sequence, 5′-UUACUUGA-3′. (**B**) An RNA pull-down assay was performed to identify the interaction between hsa-circ-0001146 and miR-26a-5p. The result showed that Bio-miR-26a-5p-WT probe can bind to hsa-circ-0001146, while Bio-miR-26a-5p-Mut probe fail to bind to miR-26a-5p. ***P<0.001 vs. Bio-miR-NC. (**C**) Analysis of the luciferase reporter assay found that miR-26a-5p mimics decreased the luciferase activity of MNAT1 WT constructors, but not that of MNAT1 MT constructors. **P<0.01 vs. WT group. (**D**) Transfection with siRNA down-regulated hsa-circ-0001146 expression in MG-63 and 143B cells. (**E**) miR-26a-5p expression was down-regulated and up-regulated by the inhibitors and mimics, respectively. hsa-circ-0001146 down-regulation and miR-26a-5p up-regulation decreased the mRNA (**F**) and protein levels (**G**) of MNAT1, while the miR-26a-5p reduction promoted MNAT1 expression. *P<0.05 and **P<0.01 vs. control group.

As described by Figure 3A, hsa-circ-0001146 is formed by the junction between 5′ and 3′ regions of *CPNE1* mRNA variant 3. hsa-circ-0001146 contains the sequences of Exon 2 and introns at both sides of Exon 2, according to information provided by three circRNAs’ websites: http://www.circbase.org/; http://202.195.183.4:8000/circrnadb/circRNADb.php, and http://gb.whu.edu.cn/CSCD/. Although hsa-circ-0001146 gene was located at chr20 from 34241449 to 34246936, the mature hsa-circ-0001146 is only composed of 329 bases. Hsa-circ-0001146 was up-regulated in OS tissues in comparison with the corresponding normal tissues (*P*<0.05, Figure 3B), while *CPNE1* mRNA expression showed no difference between the cancer and normal tissues. FISH analysis demonstrated that hsa-circ-0001146 was located in both nucleus and cytoplasm of 143B cells (Figure 3C). Bioinformatics analysis showed that miR-26a-5p can bind to both hsa-circ-0001146 and MNAT1 3′UTR at completely consistent base sequence, 5′-UUACUUGA-3′ (Figure 4A). This suggested that hsa-circ-0001146 and MNAT1 3′UTR may competitively bind to miR-26a-5p. This study performed an RNA pull-down assay to identify the interaction between hsa-circ-0001146 and miR-26a-5p. The result showed that Bio-miR-26a-5p-WT probe can bind to hsa-circ-0001146 (*P*<0.001), while Bio-miR-26a-5p-Mut probe fail to bind to miR-26a-5p (Figure 4B). Analysis of the Luciferase reporter assay found that miR-26a-5p mimics decreased the luciferase activity of MNAT1 WT constructors (*P*<0.01), but not that of MNAT1 MT constructors (Figure 4C). To further determine the regulatory effects of has-circ-0001146 and miR-26a-5p on MNAT1 expression, the present study epigenetically changed the expression of has-circ-0001146 and miR-26a-5p in OS cells. Transfection with shRNA down-regulated hsa-circ-0001146 expression in MG-63 (*P*<0.01, Figure 4D) and 143B cells (*P*<0.01). miR-26a-5p expression was down-regulated and up-regulated by the inhibitors and mimics, respectively (*P*<0.01, Figure 4E). hsa-circ-0001146 down-regulation and miR-26a-5p up-regulation decreased the mRNA and protein levels of MNAT1 (*P*<0.05 or *P*<0.01), while the miR-26a-5p reduction promoted MNAT1 expression (*P*<0.05 or *P*<0.01, Figure 4F,G).

### Has-circ-0001146/miR26a-5p/Mnat1 network regulated the growth and invasion of OS cells

**Figure 5:**
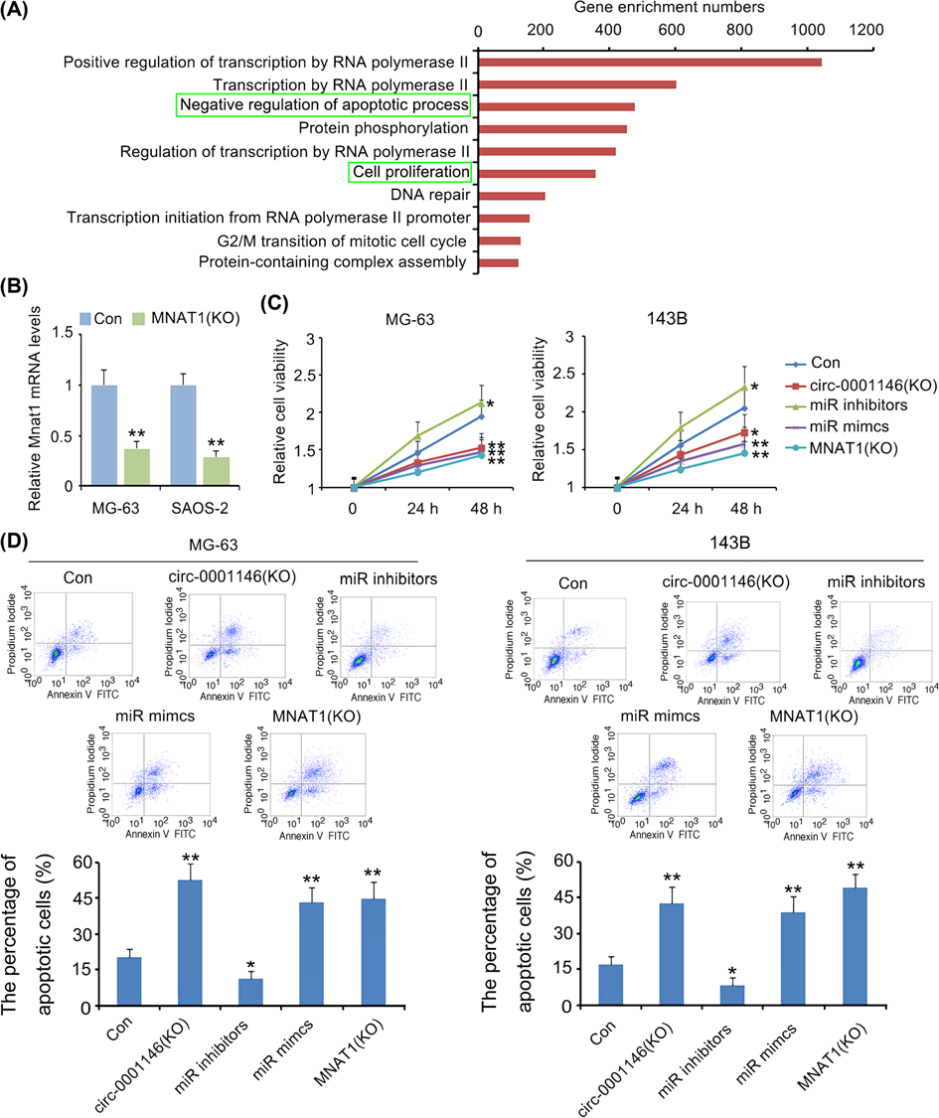
The regulatory effects of has-circ-0001146/miR26a-5p/Mnat1 network on cell viability and apoptosis (**A**) We initially analysed the genes that are co-expressed with MNAT1 genes (http://ualcan.path.uab.edu/analysis.html), as well as proteins that interact with MNAT1 protein (https://string-db.org/). Next, GO biological processes the genes and proteins were analysed using David web (https://david.ncifcrf.gov/). Results showed that MNAT1 is involved in the multiple functions such as cell apoptosis and proliferation. (**B**) As indicated by PCR assay, siRNA-MNAT1 remarkably reduced MNAT1 expression in MG-63 and 143B cells. (**C**) MG-63 and 143B cells were transfected with siRNA-hsa-circ-0001146, miR-26a-5p inhibitors and mimics, and siRNA- MNAT1, followed by cell viability measurement. Osteosarcoma cell viability was inhibited by MNAT1 and has-circ-0001146 knockdown and miR-26a-5p overexpression. However, the deficiency of miR-26a-5p enhanced the cell viability. (**D**) OS cell apoptosis rate was increased by miR-26a-5p reduction, but was decreased by the reduction of has-circ-0001146 and MNAT1 and increase of miR-26a-5p. *P<0.05 and **P<0.01 vs. control group.

We initially analysed the genes that are co-expressed with MNAT1 genes (http://ualcan.path.uab.edu/analysis.html), as well as proteins that interact with MNAT1 protein (https://string-db.org/), because these genes and proteins are very likely associated with the biological functions of MNAT1. Next, GO biological processes of the genes and proteins were analysed using David web (https://david.ncifcrf.gov/). Results showed that MNAT1 is involved in the multiple functions such as cell apoptosis and proliferation (Figure 5A). To identify the prediction, the present study knocked down the expression of MNAT1 in OS cells using siRNA-MNAT1. As indicated by PCR assay, siRNA-MNAT1 remarkably reduced MNAT1 expression in MG-63 and 143B cells (*P*<0.01, Figure 5B). OS cell viability was inhibited by MNAT1 knockdown (*P*<0.01, Figure 5C). This inhibitory effect was also observed with has-circ-0001146 knockdown and miR-26a-5p overexpression (*P*<0.05 or *P*<0.01). However, the deficiency of miR-26a-5p enhanced the cell viability (*p* < 0.05). Conversely, OS cell apoptosis rate was increased by miR-26a-5p reduction (*P*<0.05, Figure 5D), but was decreased by the reduction of has-circ-0001146 and MNAT1 and increase of miR-26a-5p (*P*<0.01).

**Figure 6:**
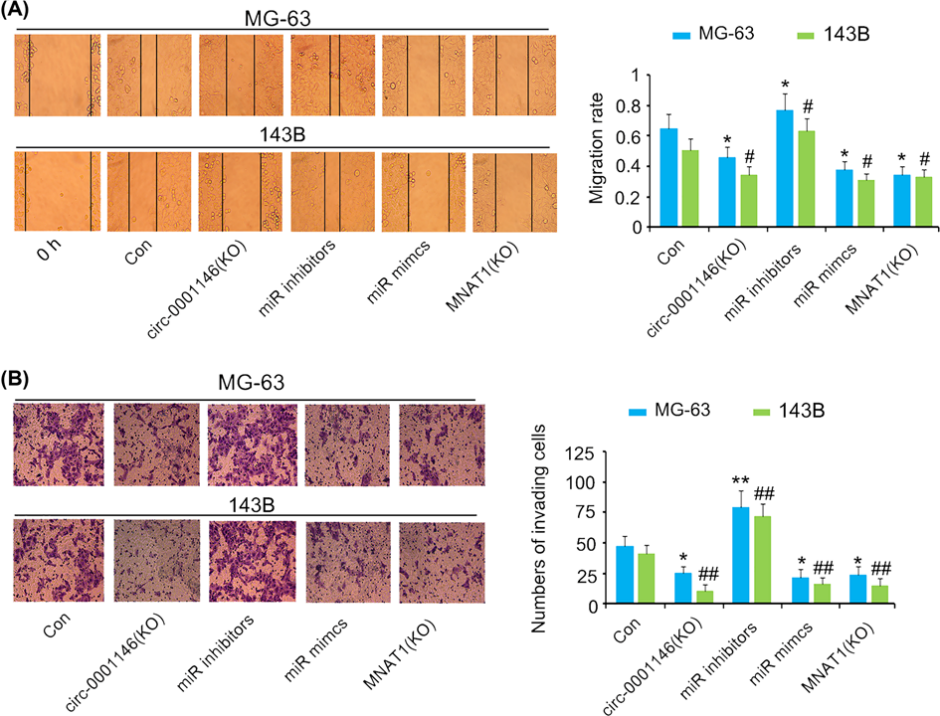
The regulatory effects of has-circ-0001146/miR26a-5p/Mnat1 network on cell migration and invasion MG-63 and 143B cells were transfected with siRNA-hsa-circ-0001146, miR-26a-5p inhibitors and mimics, and siRNA-MNAT1, followed by cell migration and invasion measurements. (**A**) Migration of MG-63 and 143B cells was enhanced after miR-26a-5p depletion, but was inhibited after the reduction of has-circ-0001146 and MNAT1 and increase of miR-26a-5p. (**B**) The deficiency of miR-26a-5p also enhanced the invasion of MG-63 and 143B cells. MG-63 and 143B cell invasion was impaired after the reduction of has-circ-0001146 and MNAT1 and increase of miR-26a-5p. *P<0.05 and **P<0.01 vs. MG-63 control cells, #*P*<0.05 and ##*P*<0.01 vs. 143B control cells.

Migration of MG-63 and 143B cells was enhanced after miR-26a-5p depletion (*P*<0.05, Figure 6A), but were inhibited after the reduction of has-circ-0001146 and MNAT1 and increase of miR-26a-5p (*P*<0.05). Similarly, the deficiency of miR-26a-5p also enhanced the invasion of MG-63 and 143B cells (*P*<0.01, Figure 6B). MG-63 and 143B cell invasion was impaired after the reduction of has-circ-0001146 and MNAT1 and increase of miR-26a-5p (*P*<0.05 or *P*<0.01).

**Figure 7:**
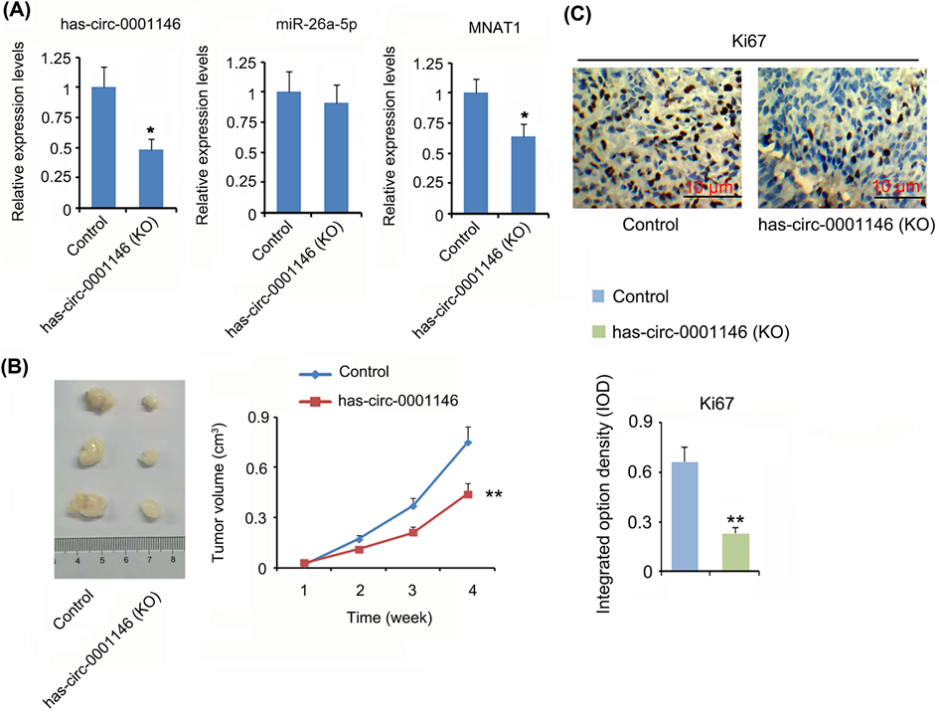
The regulatory effects of hsa-circ-0001146 on MNAT1 expression and xenograft tumor growth in nude mice (**A**) Transfection with shRNA down-regulated hsa-circ-0001146 expression in 143B tumor, which was in companied with reduction of MNAT1 expression. hsa-circ-0001146 knockdown retarded the tumor growth (**B**) and caused the down-regulation of Ki67, a cell-proliferative mark (**C**). *P<0.05 and **P<0.01 vs. control group.

Our previous study demonstrated that overexpression of MNAT1 promoted xenograft tumor growth and metastasis to lung tissues [13]. Given hsa-circ-0001146 was implicated in MNAT1 expression, the present study stably knocked down hsa-circ-0001146 expression in 143B cells to determine the effects on MNAT1 tumor-promoting actions *in vivo*. Transfection with shRNA down-regulated hsa-circ-0001146 expression in 143B tumor (*P*<0.05, Figure 7A). MNAT1 expression was also reduced with hsa-circ-0001146 knockdown (*P*<0.05). However, miR-26a-5p level was not significantly changed after hsa-circ-0001146 knockdown. hsa-circ-0001146 knockdown retarded the tumor growth (*P*<0.01, Figure 7B) and caused the down-regulation of Ki67, a cell-proliferative mark (*P*<0.01, Figure 7C).

## Discussion

The present study demonstrated higher *MNAT1* mRNA and protein expression in OS tissues than in adjacent normal tissues. However, the expression of MNAT1 was not significantly up-regulated in SARC according to TCGA databases. Higher expression of MNAT1 was associated with shorter overall survival time of SARC patients. Our previous study found that high MNAT1 expression increased the risk of pulmonary metastasis of OS cells [13]. The underlying mechanism of this is related to the activation of AKT signalling by MNAT1. In addition, MNAT1 has also been found to play important roles in pathogenesis of breast cancer and colorectal cancer [5]. The pathological effects of MNAT1 may be associated with the influence of activity on CAK and a series of CAK downstream regulators such as CDKs, p53, Octs, RARα, and PPARγ.

Bioinformatics analysis demonstrated a negative correlation between miR-26a-5p and MNAT1 and revealed a potential binding site of miR-26a-5p to 3′UTR of *MNAT1* mRNA. These data implied that *MNAT1* mRNA was a potential target of miR-26a-5p. The present study epigenetically modulated the expression of miR-26a-5p using the inhibitor and mimics in OS cells. Results showed that miR-26a-5p overexpression caused the reduction of *MNAT1* mRNA, while downregulation of miR-26a-5p increased *MNAT1* mRNA levels. The interaction between miR-26a-5p and *MNAT1* mRNA was also confirmed by a luciferase report assay. A previous report has shown the suppressive effect of miR-26a-5p on malignant behaviours of OS cells through targeting autophagy activating kinase [14]. The present study revealed that miR-26a-5p can also down-regulate MNAT1 to suppress malignant behaviours of OS cells. In addition, a few of recent studies reported that miR-26a-5p plays cancer-suppressive role in several other type of cancers, such as papillary thyroid carcinoma, colorectal cancer, breast cancer, hepatocellular carcinoma, and melanoma [15–19].

It is possible that the cancer-suppressive effect of miR-26a-5p in OS is also due to other ncRNAs such lncRNAs and circRNAs due to their miRNA-sponging effect. Wang and Sun found that reduced expression of FOXO1 increased lncRNA MALAT1 in OS. MALAT1 further sponged miR-26a-5p, thereby disrupting the cancer-suppressive effect of miR-26a-5p [20]. Similarly, the present study found that has-circ-0001146 can bind to miR-26a-5p. More importantly, the binding site (sequences) of miR-26a-5p at has-circ-0001146 is consistent to that of miR-26a-5p at *MNAT1* mRNA, suggesting the competitive binding of both has-circ-0001146 and *MNAT1* mRNA to miR-26a-5p. Given the up-regulation of has-circ-0001146 in OS according to the PCR analysis, has-circ-0001146 very likely interfered with the binding of miR-26a-5p to *MNAT1* mRNA, resulting in the abnormal up-regulation of *MNAT1* mRNA. In agreement with this hypothesis, the present study knocked down has-circ-0001146, which caused the down-regulation of *MNAT1* mRNA.

To the best of our knowledge, the role of has-circ-0001146 in cancer properties has never been studied. The present study found that has-circ-0001146 knockdown inhibited OS cell viability, migration and invasion, with increased apoptosis rates. These data suggested a cancer-promoting role of has-circ-0001146 in OS. Since the present study identified the existence of the has-circ-0001146/miR26a-5p/Mnat1 network in OS, the cancer promoting effect of has-circ-0001146 in OS is potentially associated with the interference of miR26a-5p targeting Mnat1. Further study is warranted to systemically investigate the cancer promoting effect of has-circ-0001146 in OS.

We found that miR-26a-5p expression level was not changed after the knockdown of has-circa-0001146 in the animal study. The possible reason is that the knockdown of has-circa-0001146 caused the reduction of miR-26a-5p binding to has-circa-0001146, but increased the connection of miR-26a-5p with MNAT1 mRNA. Both miR-26a-5p and MNAT1 are degraded after stimulating RNA-induced silencing. However, the knockdown of has-circa-0001146 might also decreased the degradation of miR-26a-5p that originally targets has-circa-0001146.

In summary, the present study of the has-circ-0001146/miR26a-5p/Mnat1 network revealed the role of the network in significantly influencing the OS hallmarks, including cell viability, apoptosis, migration, and invasion. In addition, the overexpression of has-circ-0001146 is likely an important cause of the up-regulation of Mnat1 in OS. Therefore, the present study has further added to our understanding of the pathogenesis of OS.

## Abbreviations

CAK: cyclin-dependent kinase-activating kinase
circRNA: circular RNA
miRNA: microRNA
MTA1: menage a trois 1
ncRNA: non-coding RNA
OS: osteosarcoma
PPARγ: peroxisome proliferator-activated receptor γ
RARα: retinoic acid receptor α
